# First Record of *Alternaria pogostemonis*: A Novel Species Causing Leaf Spots in *Pogostemon cablin*

**DOI:** 10.3390/pathogens11101105

**Published:** 2022-09-27

**Authors:** Mei Luo, Minping Zhao, Yinghua Huang, Jiawei Liu, Qiurong Huang, Yongxin Shu, Zhangyong Dong

**Affiliations:** 1 Innovative Institute for Plant Health, Zhongkai University of Agriculture and Engineering, Guangzhou 510225, China; 2 Key Laboratory of Fruit and Vegetable Green Prevention and Control in South-China, Ministry of Agriculture and Rural Affairs, Guangzhou 510225, China

**Keywords:** *Alternaria*, new host record, pathogenicity, phylogeny

## Abstract

*Pogostemon cablin* (Lamiaceae) is a component of traditional medicines in Southern China. The identification of *P*. *cablin* pathogens is essential for the production and development of this industry. During 2019–2020, a leaf spot on *P*. *cablin* was observed in Zhanjiang, Guangdong Province. The pathogen of the leaf spot was isolated and identified using morphological and phylogenetic methods. Phylogenetic analysis was performed using the internal transcribed spacer (ITS) region, glyceraldehyde-3-phosphate dehydrogenase (*gapdh*), RNA polymerase II (*rpb2*), translation extension factor 1-alpha (*tef1*), and Alternaria major allergen 1 (*Alt-a1*) genes. Based on phylogenetic and morphological studies, this was confirmed to be a novel species of *Alternaria pogostemonis*, with description and illustrations presented. The pathogenicity test of *A*. *pogostemon* was verified by Koch’s postulates as causing leaf spot disease. This is the first report of leaf spot disease in *P. cablin* caused by the *Alternaria* species. This study contributes to the knowledge of *P. cablin* leaf spot diseases.

## 1. Introduction

*Pogostemon cablin* (Blanco) Benth, family Lamiaceae, originates from Malaysia and Indonesia. *Pogostemon cablin* is distributed extensively across south-east Asia, including China, India, Indonesia, Sri Lanka, the Philippines, and Malaysia [1,2,3]. Patchouli is well known for its aromatic properties as an essential oil and perfume [4], and also for its medicinal properties [5]. Notably, *P. cablin* is a traditional medicinal plant in China, and is widely cultivated in Guangdong, Guangxi, Hainan, Fujian, and Taiwan, as well as other places in China [6]. The stems and leaves can be used for medicinal purposes. Clinically, it is widely used to treat heat exhaustion, chest distress, abdominal pain, vomiting, and diarrhea [5,7]. It is an essential raw material in over 30 Chinese patent medicines such as the “Huoxiang Zhengqi Pill” and “antiviral oral liquid.”

Various pathogens, including bacteria such as *Ralstonia solanacearum* [8], plant nematodes such as *Meloidogyne incognita* [9,10], and viruses such as *P. cablin* yellow mosaic virus (PaYMV) [5] have been reported to infect *P*. *cablin.* However, few fungal diseases have been reported in this host. Chen et al. [11] reported that *Corynespora cassiicola* caused leaf spots. Zeng et al. [12] observed a *Phomopsis* leaf spot caused by *Diaporthe arecae* in Guangzhou, China. Dong et al. [13] identified a novel taxon of *Stagonosporopsis pogostemonis* causing leaf spots and stem blight on *P. cablin*.

*Alternaria*, with *Alternaria tenuis* as the type species, was introduced by Nees (1817). There are currently 797 accepted specific epithets for *Alternaria* in the Index Fungorum and 702 specific epithets in the species Fungorum (July 2022). Wijayawardene et al. [14] reported that *Alternaria* contains 366 accepted and recognizable species. *Alternaria* black spot, blight disease, and seed-borne pathogens are major pathogens distributed worldwide on cruciferous crops and other economically relevant plants that cause considerable yield losses [15,16,17].

The cultivation of *P*. *cablin* is simple and primarily relies on wireless cutting propagation. The long-term asexual propagation has resulted in single varieties and a narrow genetic base of *P*. *cablin*, resulting in germplasm degradation and decreased disease resistance. Therefore, identifying pathogens of *P*. *cablin* is significant for the cultivation and development of the *P*. *cablin* industry.

To this end, we observed a new leaf disease in the *P. cablin* fields in Zhanjiang City of Guangdong Province, China, between 2019–2020. Samples were collected and the putative pathogen was isolated. We aimed to identify the fungal groups that cause leaf spot disease in *P*. *cablin* by combining morphological characteristics and phylogenetic analysis. Further, we evaluated whether the pathogenicity of the putative pathogen conforms to Koch’s hypothesis.

## 2. Materials and Methods

### 2.1. Sample Collection and Pathogen Isolation

Diseased *P. cablin* were collected from the fields in Zhanjiang City, Guangdong Province, China (E 110°3′, N 21°2′) from the spring of 2019 to the summer of 2020. Images were captured (Nikon D300s, Japan), and the time, location, latitude, longitude, and species of the sampled plants were recorded.

The collected samples were washed with running tap water for several minutes and subsequently with sterile water. The diseased leaves were cut with a sterile scalpel into small pieces (approximately 0.5 × 0.5 cm^2^) between the diseased spots and the healthy part. The surface was disinfected with 75% alcohol for 10 s and 2.5% NaClO for 15 s. After disinfection, the plant tissues were washed three times for 30 s with sterile water. Five pieces were dried on sterile filter paper and then placed on a 9-mm potato dextrose agar (PDA) plate containing a final concentration of 100 mg/L streptomycin sulfate.

After being incubated in the dark at 28 °C for 2–3 days, the individual mycelium tips were transferred to a PDA plate. Then they were purified thrice by hyphal tip isolations. Strains and plant samples were deposited in the Culture Collection of Zhongkai University of Agriculture and Engineering (ZHKUCC).

### 2.2. DNA Extraction and PCR Amplification

Total genomic DNA was extracted from fungi cultured in PDA for seven days. Fresh hyphae were collected and DNA was extracted by the modified CTAB method [18]. Molecular amplification of the following regions was performed: internal transcribed spacer (ITS) regions, glyceraldehyde-3-phosphate dehydrogenase (*gapdh*), RNA polymerase II (*rpb2*), translation extension factor 1-alpha (*tef1*), and Alternaria major allergen 1 (*Alt-a1*) genes (Table 1). The total reaction solution in the PCR amplification instrument was 25 μL, containing 1 µL genomic DNA, 1 µL of each forward/reverse primer (10 μm), 12.5 μL I-5™ 2× Easy *Taq* PCR Supermix (+dye) (Transgen Biotech, China), and 9.5 μL deionized distilled water (ddH_2_O). The thermal cycling conditions used for PCR amplification are listed in Table 1. The positive amplified sequences were sequenced by Guangzhou Tianyi Technology Co., Ltd. (Guangzhou, China).

### 2.3. Phylogenetic Analysis

Sequence quality was assured by validating chromatograms using BioEdit v5. The resulting sequences were checked against the National Center for Biotechnology Information (NCBI) search engine GenBank BLASTn (https://blast.ncbi.nlm.nih.gov/Blast.cgi, accessed on 12 January 2022). According to the BLAST results, the ITS, *gapdh*, *rpb2*, *tef1-α*, and *Alt-a1* sequences obtained in this study were closely related to *Alternaria.* Relevant sequence data were downloaded using Genbank. The maximum likelihood (ML) in RAxML [24] was run for all the *Alternaria* species. After confirming that the strains from our study belonged to the *A*. *alternaria* species complex (AALSC), phylogenetic analysis was performed with these strains. The individual sequence dataset was aligned using MAFFT v.7, http://mafft.cbrc.jp/alignment/server accessed on 1 July 2022), and improved manually using BioEdit v5 [25] as required. Subsequently, the aligned datasets were concatenated manually. All sequences obtained in this study are deposited in GenBank (Table S1). Phylogenetic analyses were performed by ML in RAxML [24] and Bayesian analyses (BI) in Mr Bayes v. 3.0b4 [26].

The maximum likelihood analyses were performed using RAxML-HPC2 on XSEDE (8.2.8) [27] on the CIPRES Science Gateway platform [28]. The best model of evolution for each gene was determined by MrModeltest v. 2.2. The GTR + I + G evolutionary model was employed with 1000 non-parametric bootstrapping iterations. MrModeltest v. 2.3 [29] was used to identify the evolutionary models for each locus used in Bayesian analysis. The Markov Chain Monte Carlo sampling (MCMC) analysis was conducted with four simultaneous Markov chains. These were run for 1,000,000 generations, sampling the trees at every 100^th^ generation. From the 10,000 trees obtained, the first 2000 representing the burn-in phase were discarded. The remaining 8000 trees were used to calculate posterior probabilities in a majority rule consensus tree. The constructed phylogenetic tree was visualized in FigTree v1.4.2 and edited in Adobe Illustrator CS6.

### 2.4. Morphological Description

The strain was cultured on PDA, oatmeal agar (OA), and malt extract agar (MEA) media. The macroscopic morphological characteristics were evaluated. The culture characters and morphology of the colonies cultured with PDA were observed in the dark at 28 °C. Pycnidia were cut by a freezing sliding microtome (Bio-Key science and technology Co., Ltd., LEICA CM1860, Weztlar, Germany) for imaging and subsequent measurements. Conidiomata were visualized using SteREO Discovery.V20 (Zeiss, Germany). Digital images of the microstructure (shape, size, and color) were captured using a Nikon Eclipse 80i microscope (Nikon, Tokyo, Japan). The conidia length and width of 30 spores were measured by NIS-element BR3.2. The mean value and standard deviation (SD) were calculated using Microsoft Excel (Microsoft, Redmond, WA, USA).

### 2.5. Pathogenicity Tests

Pathogenicity tests using *P*. *cablin* seedlings were conducted in the greenhouse using the mycelial plug method and suspension inoculation. Inoculated plants were kept in the greenhouse (25 °C) with artificial lighting (14 h period of supplementary lighting/10 h dark). Six *P. cablin* leaves from six plants were picked for each method. The surfaces of the leaves were first wiped clean with wet sterile cotton and disinfected with 75% alcohol. The leaves were then wiped three times with sterile wet cotton. Some of the leaves were punctured with a sterilized No. 3 insect needle. The fungi plate was beaten into fungus blocks with a 5 mm diameter. The fungus blocks were placed on the injured leaves and covered with a film. A 5-mm PDA plate was used as a control. The mycelium was put into a 150 mL PD medium and shaken for five to seven days to prepare the mycelium suspension. The 10% mycelial suspension (10 mg/100 mL [volume]) was crushed using a juice extractor as per Dong et al. [15] and sprayed on the leaves and stems with sterile cotton. The leaves and stems were then covered with wet cotton and sealed with Parafilm or bagged for moisturizing for 24–48 h. The *P*. *cablin* leaves and seedlings were observed every day. After the onset of the disease, the pathogen was isolated to confirm Koch’s postulates.

## 3. Results

### 3.1. Field Symptoms

**Figure 1:**
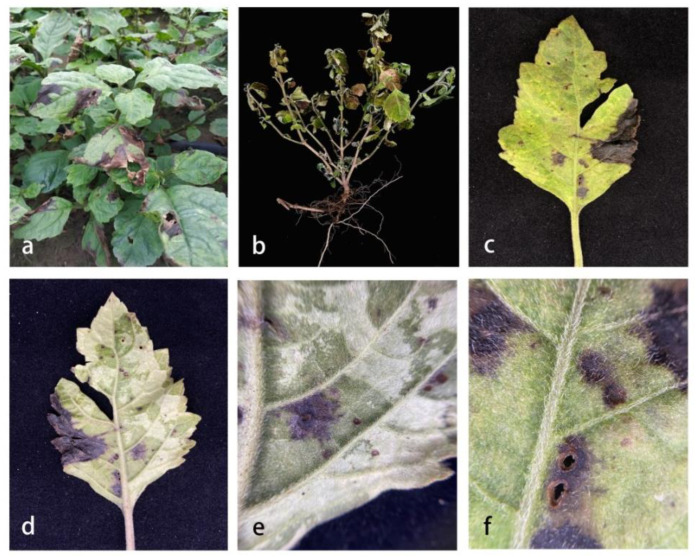
Symptoms of leaf spots caused by isolates in *Pogostemon cablin*. (**a**) Symptom in the field; (**b**) Infected plants; (**c**) The positive surface of an infected leaf; (**d**) The opposite side of an infected leaf; (**e**,**f**) A local area of an infected leaf.

The disease incidence was approximately 15–30% at high temperatures above 30 °C, and high humidity in the summer. Yellow-brown round spots initially appeared on the leaves and were round or irregularly round and brown in the middle stage. In the later stage, several spots connected, which led to the scorched shedding of the spots. Some leaves perforated from the center of the disease spot, and eventually the whole leaf became perforated and worthless (Figure 1).

### 3.2. Morphological and Molecular Characterization

**Figure 2:**
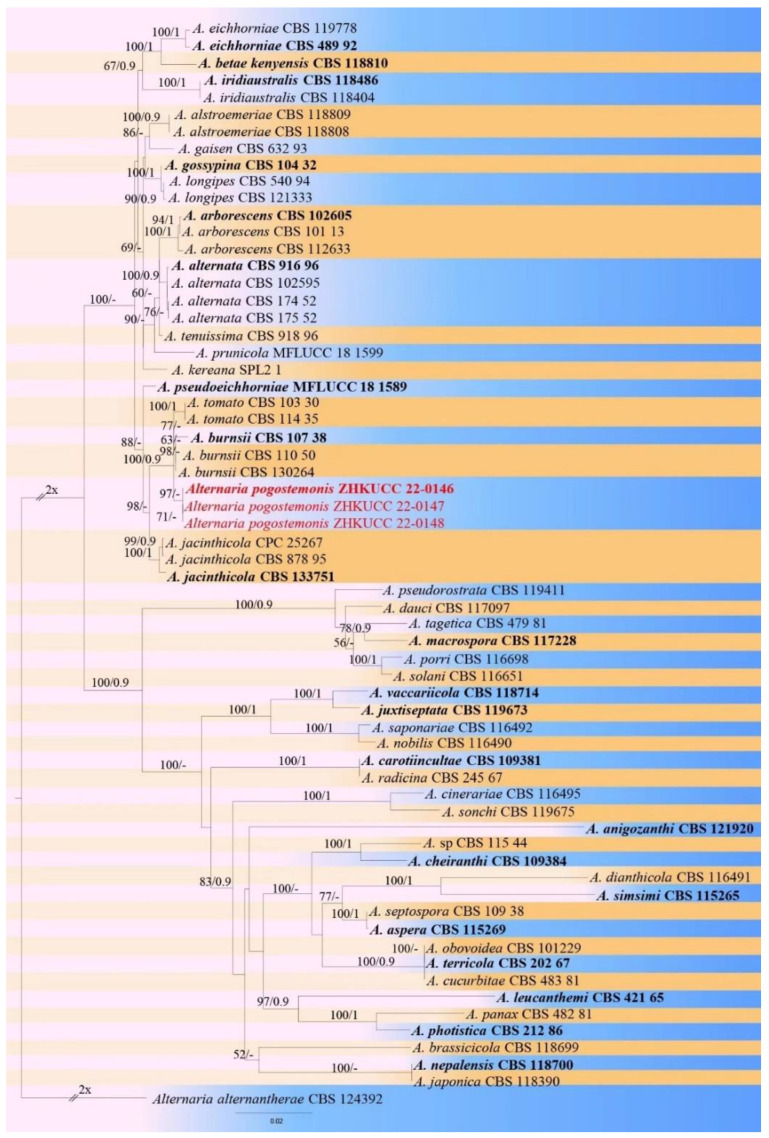
The best scoring maximum likelihood (ML) tree obtained from a heuristic search of the combined ITS, *gapd*, *rpb2*, *tef 1-α*, and *Alt-a1* sequence alignment of the *Alternaria alternaria* complex species. Bootstrap support values equal to greater than 50% in ML and posterior probabilities (PPs) equal or greater than 0.90 are shown as ML/PPs above the respective node. *Alternaria alternantherae* (CBS 124392) are used as outgroup taxa. Ex-type strains are in bold and isolates belonging to this study are in red.

**Figure 3:**
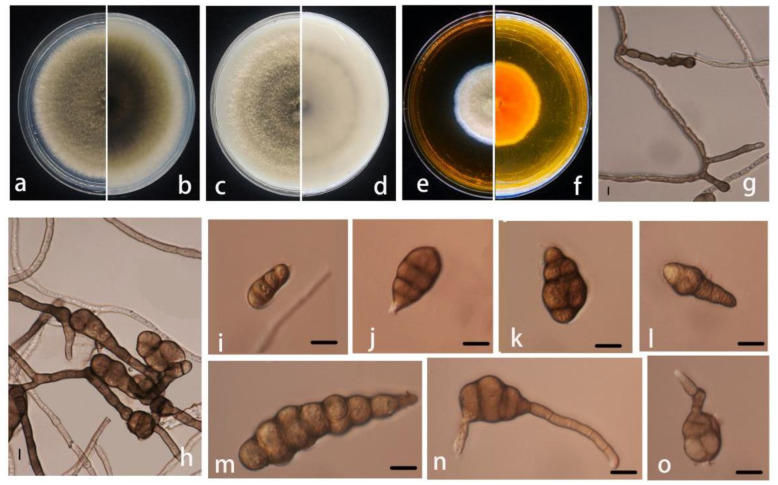
Morphological characteristics of *Alternaria pogostemonis* (ZHKUCC 22-0146). (**a**,**b**) Front and reverse view on PDA after 5 days at 28 °C; (**c**,**d**) Front and reverse view on MEA after 7 days at 28 °C; (**e**,**f**) Front and reverse view on OA after 7 days at 28 °C; (**g**,**h**) Sporulation pattern; (**i**–**o**) Conidia morphology bars: (**g**–**o**) = 10 μm.

Three isolates were obtained in this study. These were confirmed to be morphologically similar to species of *Alternaria*. In addition, BLASTn analysis of the ITS region indicated their highest sequence identity to fungi of the genus *Alternaria*. The combined sequence data set comprised three *Alternaria* isolates from this study and 63 reference sequences. The resulting tree was rooted with *A*. *alternantherae* (CBS 124392). The tree topology of the ML analysis was similar to the PPs (Figure 2). The best scoring RAxML tree with a final likelihood value of -15189.796179 is presented in Figure 2. The matrix had 885 distinct alignment patterns, with 7.75% of undetermined characters or gaps. Estimated base frequencies were as follows: A = 0.240574, C = 0.282504, G = 0.244359, T = 0.232563; substitution rates AC = 1.169297, AG = 3.484578, AT = 1.120356, CG = 0.748282, CT = 6.861660, GT = 1.000000; gamma distribution shape parameter ɑ = 0.313383. Isolates obtained in this study developed a clade together with *A. burnsii* and *A. tomato* with 100% ML and 0.90 PPs. Therefore, we compared the morphology and pairwise nucleotide differences among strains isolated in this study with *A. burnsii* and *A. tomato*. Based on molecular and morphological evidence, isolates obtained in this study (ZHKUCC 22-0146, ZHKUCC 22-0147, and ZHKUCC 22-0148) were identified as a novel species. The species description is as follows:

***Alternaria pogostemonis*** M. Luo, M.P. Zhao, and Z.Y. Dong, sp. *nov*.;
Index Fungorum number: IF554928 (Figure 3).
Etymology: In reference to the host genus name *Pogostemon*;
Holotype: ZHKUCC 22-0146;

Pathogenic on *Pogostemon cablin* leaves. Sexual morphology: Not observed. Asexual morphology: Hyphae surface covered with dense hyphae, subhyaline, branched, smooth, warty, septum, 1–3 μm wide. *Conidiophores* solitary or branched, brown, many septate, and terminal meristematic locus simple. Conidia 17–77 × 9–22 μm ($\bar{x}$ = 33 × 14 μm, n = 50), scattered, 20 or more single or branch chains of conidium, elliptic or ovate, light brown to brown, brown conidium to transparent, no branch is an inverted stick, inverted pear-shaped, ovoid, or oblong, conical or cylindrical short beak, brown to brown, shape, size differed, usually with 2–7 transverse septa and 0–5 longitudinal septa.

Culture characteristics: Colonies on PDA and OA media reach 85 mm diameter after 7 days at 25 °C. The colony on PDA was circular, entire-edged, flat, floccose to woolly, first cotton-like, then generally gray-brown from the center outward from gray to expand the edge of white. Brown on the back.

Material examined: Zhanjiang, Guangdong Province, China, isolated from diseased leaves of *Pogostemon cablin*, April 2020, by Y. Huang and Y. Shu (dried cultures ZHKU 22-0082, holotype, ex-type culture ZHKUCC 22-0146 and ex-paratype ZHKUCC 21-0147 and ZHKUCC 21-0148).

Notes: The three strains (ZHKUCC 22-0146–0148) obtained in this study constituted a monophyletic clade with *A. burnsii* and *A. tomato* with a 100% maximum likelihood bootstrap and Bayesian posterior probability value of 0.90. When compared, the genes between *A. pogostemonis* and *A. burnsii* exhibited 1.25% differences (240 nucleotides) in *tef1*, 0.35% differences (576 nucleotides) in *gapdh* and 0.2% differences (471 nucleotides) in *Alt-a1*. Comparison between *A. pogostemonis* and *A. tomato* revealed 1.7% differences (240 nucleotides) in *tef1*, 0.35% differences (576 nucleotides) in *gapdh*, 0.64% differences (471 nucleotides) in *Alt-a1*, and 0.40% differences (753 nucleotides) in *rpb2*. *Alternaria pogostemonis* in our study developed gray to gray-brown pigment on PDA. In contrast, other studies suggest that *A. burnsii* [15,30,31] and *A. tomato* [31] have no pigment in PDA. Furthermore, *A. pogostemonis* developed much larger spores (Table 2). Thus, based on the phylogeny and morphology, we introduce this species as a new *Alternaria* species causing disease in *P*. *cablin*.

### 3.3. Disease Symptoms and Pathogenicity Tests

**Figure 4:**
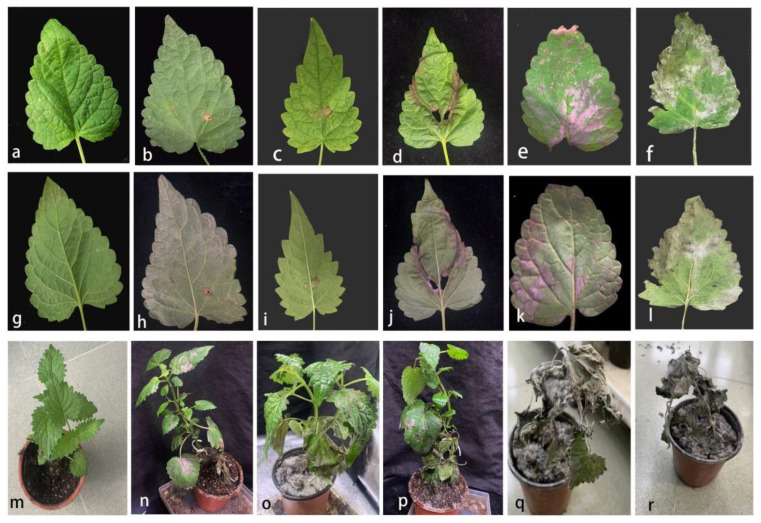
Pathogenicity study of *Alternaria pogostemonis*. (**a**,**g**) The front and back side of leaf inoculated of the control leave; (**b**,**h**) Front and back of leaf at the beginning of needling experiment; (**c**,**i**) Front and back of leaf in the middle of needling experiment; (**d**,**j**) Front and back of leaf in late needling experiment; (**e**,**k**) Front and back of leaves in the early stage of mycelium suspension experiment; (**f**,**l**) Front and back of leaves at late stage of mycelium suspension experiment; (**m**) Control plant; (**n**–**r**) Early, middle, late stage of mycelium suspension experiment.

Both the mycelial plug and suspension methods were employed. On days 3 and 4 after inoculation, leaf plaques appeared on the injured young leaves. Symptoms appeared on the old or uninjured leaves 5–7 days after inoculation. Initial symptoms were minor; however, leaf tissue eventually turned necrotic, expanding from the initial round plaque to the periphery. Some even perforated from the center. Subsequently, the part of mycelium in contact with the leaf began to dry and fall. The symptoms usually develop at the tip or margin (Figure 4b–f,h–l). Under high humidity conditions, some diseased spots appeared on the leaves on the fifth day, and the severely diseased leaves withered and fell off after seven days. After seven days of incubation, the stems first browned on the surface, and then became dry and shriveled (Figure 4n–r). No disease symptoms developed on any of the controls (Figure 4a,g). Finally, fungal isolates were isolated from the infected leaves, and the phenotype and phylogeny were compared to verify Koch’s hypothesis.

## 4. Discussion

A novel leaf spot disease was isolated from *P. cablin* in Zhanjiang city, Guangdong Province, in May 2020, and the pathogen was identified as *A.* *pogostemonis*. The disease is initially characterized by yellowish-brown round spots on the leaves and by round or irregular round and brown spots in the middle stage. In the later stage, the disease spots expand and the leaves wither and fall. During the isolation, we also isolated other fungi together such as *Colletotrichum*, *Diaporthe*, *Epicoccum*, *Nigrospora*, and *Stagonosporopsis pogostemonis* [13]. Both *S. pogostemonis* [13] and *A.* *pogostemonis* were verified as pathogens during the pathogenicity tests. Whether there are other strains or they cause a compound infection requires further investigation.

*Alternaria*, consisting of hundreds of species, is considered one of the most critical phytopathogens affecting plant tissues, including leaves, cereal grains, fruits, and vegetables [32,33,34,35]. It has been recorded as a critical fungal pathogen because of its worldwide occurrence on various hosts [32,33,34,35].

Distinguishing the *A. burnsii*–*A. tomato* species complex based on the evolutionary tree alone is difficult [36,37]. These two species have few differences in their gene loci [36,37]. On the evolutionary tree, the strains in our study constituted a monophyletic clade with *A. burnsii* and *A. tomato*. The identified strain had more sequence similarity with *A. burnsii* and was significantly different from *A. tomato*. Further, the morphological characteristics of the colonies are varied; *Alternaria* *pogostemonis* developed much larger spores. More strains and genes must be analyzed to confirm the relationship among the *A. burnsii*–*A. tomato*–*A.* *pogostemonis* species complex.

*Alternaria* has strong adaptability to different environments and hosts. They can be plant pathogens [36,37], saprobes [37], and endophytes [38]. They have also been isolated from soil, atmosphere, and indoor environments [37,39]. The pathogenicity tests revealed that the strain in our study could induce spot symptoms on both wounded and unwounded leaves. However, the wounded leaves developed disease spots much more rapidly, with severe symptoms.

To our knowledge, this is the first report of the genus *Alternaria* causing leaf spots in *P*. *cablin*. This study represents the first detailed investigation of the morphology, phylogeny, and pathogenicity of *Alternaria* species causing *P. cablin* leaf spots in China. Species identification and confirmation of pathogenicity are critical to developing effective control measures [40]. Therefore, further studies on their biological characteristics, suitable fungicides, and their impact on *P*. *cablin* cultivation are warranted.

## Figures and Tables

**Table 1:** Gene regions and respective primer pairs used in the study.

Gene	Primer	Primer DNA Sequence (5′–3′)	Reference
ITS	ITS 4	TCCTCCGCTTATTGATATGC	[19]
	ITS5	GGAAGTAAAAGTCGTAACAAGG	
*gapdh*	gpd1	GCCAAGCAGTGTTGTGC	[20]
	gpd2	TCCTCCGCTTATTGATATGC	
*rpb2*	fRPB2-5F	GAYGAYMGWGATCAYTTYGG	[21]
	fRPB2-7cR	CCCATRGCTTGTYYRCCCAT	
*tef1-α*	TEF1-728F	CATCGAGAAGTTCGAGAAGG	[22]
	TEF1-986R	TACTTG AAGGAACCCTTACC	
*Alt-a1*	Alt-F	ATGCAGTTCACCACCATCGC	[23]
	Alt-R	ACGAGGGTGAYGTAGGCGTC	

**Table 2:** Comparison of conidial morphological characteristics between *Alternaria pogostemonis* sp. nov. and its phylogenetically related species.

Species	Conidia				Pigment in PDA	References
	Size (um)	Shape	Septa			
			Transverse	Longitudinal		
*A. burnsii*	16~60 (90) × 6.5~14 (~16)	Long ellipsoid, obclavate or ovoid	2~6 (11)	0~2 (~4)	None	[15]
*A. burnsii*	25.5~105 × 8.4~20	Obovate	4~9	0~4	No report	[30]
*A. burnsii*	30~50 × 9~13	Ovoid to ellipsoid	5~8	1~5	None	[31]
*A. tomato*	30~50 × 10~13	Narrow-ovoid	6~9	1 (~2)	None	[31]
*A. pogostemonis*	17–77 × 9–22 μm ($\bar{x}$ = 3 3 × 14 μm, n = 50)	Long ellipsoid, obclavate or ovoid	2~7	0~5	grey to grey brown	This study

## Data Availability

The sequence data generated in this study are deposited in NCBI GenBank (https://www.ncbi.nlm.nih.gov/genbank, accessed on 31 July 2022). All accession numbers are given in Table S1.

## Supplementary Materials

The following supporting information can be downloaded at: https://www.mdpi.com/article/10.3390/pathogens11101105/s1, Table S1: Strains used for the phylogenetic analyses in this study and their GenBank accession Numbers.
