# Supplementary material for: First Record of Alternaria pogostemonis: A Novel Species Causing Leaf Spots in Pogostemon cablin
Source: Pathogens. 2022 Sep 27;11(10):1105. doi: 10.3390/pathogens11101105 (PMC9607968; doi:10.3390/pathogens11101105)
Supplement: Supplementary file 1 [file pathogens-11-01105-s001.zip › pathogens-1908365-supplementary.pdf]

pathogens

Table S1. Strains used for the phylogenetic analyses in this study and their GenBank accession Numbers

Name	Culture Collection number[1]	GenBank accession numbers[2]				
		ITS	*gapdh*	*rpb2*	*tef1-α*	*Alt-a 1*
Alternaria alstroemeriae	CBS 118808	KP124296	KP124153	KP124764	KP125071	KP123845
Alternaria alstroemeriae	CBS 118809	KP124297	KP124154	KP124765	KP125072	-
Alternaria alternata	CBS 174.52	KC584228	KC584152	DQ677964	KC584704	KP123856
Alternaria alternata	CBS 916.96 [T]	AF347031	AY278808	KC584375	KC584634	AY563301
Alternaria alternata	CBS 102595	FJ266476	AY562411	KC584408	KC584666	AY563306
Alternaria alternata	JZB3180002	MH827031	MH853645	MH853718	MH853703	MH853692
Alternaria anigozanthi	CBS 121920 [T]	KC584180	KC584097	KC584376	KC584635	-
Alternaria arborescens	CBS 102605 [T]	AF347033	AY278810	KC584377	KC584636	AY563303
Alternaria arborescens	CBS 101.13	KP124392	KP124244	KP124862	KP125170	KP123940
Alternaria arborescens	CBS 112633	KP124400	KP124252	KP124870	KP125178	KP123947
Alternaria aspera	CBS 115269 [T]	KC584242	KC584166	KC584474	KC584734	KF533899
Alternaria betae-kenyensis	CBS 118810 [T]	KP124419	KP124270	KP124888	KP125197	KP123966
Alternaria brassicicola	CBS 118699	JX499031	KC584103	KC584383	KC584642	-
Alternaria burnsii	CBS 108.27	KC584236	KC584162	KC584468	KC584727	KP123850
Alternaria burnsii	CBS 107.38 [T]	KP124420	JQ646305	KP124889	KP125198	KP123967
Alternaria burnsii	YZU 191003	MN656136	MN718662	MN656154	MN656146	MN656141
Alternaria carotiincultae	CBS 109381 [T]	KC584188	KC584106	KC584386	KC584645	-

Alternaria cheiranthi	CBS 109384	AF229457	KC584107	KC584387	KC584646	JQ905106
Alternaria cinerariae	CBS 116495	KC584190	KC584109	KC584389	KC584648	-
Alternaria cucurbitae	CBS 483.81	FJ266483	AY562418	KC584483	KC584743	-
Alternaria dauci	CBS 117097	KC584192	KC584111	KC584392	KC584651	KJ718678
Alternaria dianthicola	CBS 116491	KC584194	KC584113	KC584394	KC584653	-
Alternaria eichhorniae	CBS 489.92 T	KC146356	KP124276	KP124895	KP125204	KP123973
Alternaria eichhorniae	CBS 119778	KP124426	KP124277	KP124896	KP125205	-
Alternaria gaisen	CBS 632.93	KC584197	KC584116	KC584399	KC584658	KP123974
Alternaria gaisen	CBS 118488	KP124427	KP124278	KP125206	KP124897	KP123975
Alternaria gossypina	CBS 100.23	KP124429	KP124280	KP124899	KP125208	KP123977
Alternaria gossypina	CBS 104.32 T	KP124430	JQ646312	KP124900	KP125209	JQ646395
Alternaria gypsophilae	CBS 107.41 T	KC584199	KC584118	KC584401	KC584660	JQ646304
Alternaria iridiaustralis	CBS 118486 T	KP124435	KP124284	KP124905	KP125214	KP123981
Alternaria iridiaustralis	CBS 118404	KP124434	KP124283	KP124904	KP125213	KP123980
Alternaria jacinthicola	CBS 133751 T	KP124438	KP124287	KP124908	KP125217	KP123984
Alternaria jacinthicola	CPC 25267	KP124439	KP124288	KP124909	KP125218	KP123985
Alternaria japonica	CBS 118390	KC584201	KC584121	KC584405	KC584663	-
Alternaria juxtiseptata	CBS 119673 T	KC584202	KC584122	KC584406	KC584664	-
Alternaria leucanthemi	CBS 421.65 T	KC584240	KC584164	KC584472	KC584732	-
Alternaria longipes	CBS 540.94	AY278835	AY278811	KC584409	KC584667	AY563304
Alternaria longipes	CBS 917.96	KP124442	KP124291	KP124912	KP125226	KP123988

Alternaria longipes	CBS 121333	KP124444	KP124293	KP124914	KP125223	KP123990
Alternaria macrospora	CBS 117228 [T]	KC584204	KC584124	KC584410	KC584668	KJ718702
Alternaria nepalensis	CBS 118700 [T]	KC584207	KC584126	KC584414	KC584672	-
Alternaria nobilis	CBS 116490	KC584208	KC584127	KC584415	KC584673	JQ646385
Alternaria obovoidea	CBS 101229	FJ266487	FJ266498	KC584485	KC584745	FJ266513
Alternaria panax	CBS 482.81	KC584209	KC584128	KC584417	KC584675	-
Alternaria perpunctulata	CBS 115267 [T]	KC584210	KC584129	KC584418	KC584676	JQ905111
Alternaria photistica	CBS 212.86 [T]	KC584212	KC584131	KC584420	KC584678	-
Alternaria porri	CBS 116698	DQ323700	KC584132	KC584421	KC584679	KJ718726
Alternaria pogostemonis	ZHKUCC 22-0146	OP120855	OP160290	OP160284	OP160281	OP160287
Alternaria pogostemonis	ZHKUCC 22-0147	OP120856	OP160291	OP160285	OP160282	OP160288
Alternaria pogostemonis	ZHKUCC 22-0148	OP120857	OP160292	OP160286	OP160283	OP160289
Alternaria prunicola	MFLUCC 18–1598	MH827032	MH853646	MH853719	MH853704	MH853693
Alternaria prunicola	MFLUCC 18–1596	MH827033	MH853647	MH853720	MH853705	MH853694
Alternaria prunicola	JZB3180005	MH827034	MH853648	MH853721	MH853706	MH853695
Alternaria prunicola	JZB3180006	MH827035	MH853649	MH853722	MH853707	MH853696
Alternaria prunicola	MFLUCC 18–1597 [T]	MH827036	MH853650	MH853723	MH853708	MH853697
Alternaria prunicola	MFLUCC 18–1599	MH827037	MH853651	MH853724	MH853709	MH853698
Alternaria prunicola	JZB3180013	MH827042	MH853656	MH853729	MH853714	-

Alternaria pseudoeichhorniae	MFLUCC 18–1589 T	MH827030	MH853644	MH853717	MH853702	-
Alternaria pseudorostrata	CBS 119411 T	JN383483	AY562406	KC584422	KC584680	-
Alternaria radicina	CBS 245.67 T	KC584213	KC584133	KC584423	KC584681	FN689405
Alternaria saponariae	CBS 116492	KC584215	KC584135	KC584425	KC584683	-
Alternaria septospora	CBS 109.38	FJ266489	FJ266500	KC584487	KC584747	FJ266515
Alternaria simsimi	CBS 115265 T	JF780937	KC584137	KC584428	KC584686	JQ905110
Alternaria solani	CBS 116651	KC584217	KC584139	KC584430	KC584688	-
Alternaria sonchi	CBS 119675	KC584220	KC584142	KC584433	KC584691	-
Alternaria sp.	CBS 115.44	KC584214	KC584134	KC584424	KC584682	-
Alternaria tagetica	CBS 479.81	KC584221	KC584143	KC584434	KC584692	KJ718761
Alternaria tenuissima	CBS 918.96	AF347032	AY278809	KC584435	KC584693	AY563302
Alternaria terricola	CBS 202.67 T	FJ266490	KC584177	KC584490	KC584750	FJ266516
Alternaria tomato	CBS 103.30	KP124445	KP124294	KP124915	KP125224	KP123991
Alternaria tomato	CBS 114.35	KP124446	KP124295	KP124916	KP125225	KP123992
Alternaria vaccariicola	CBS 118714 T	KC584224	KC584147	KC584439	KC584697	-
Alternaria alternantherae	CBS 124392	KC584179	KC584096	KC584374	KC584633	KP123846
Stemphylium botryosum	ATCC 42170 T	AF229481	AY278820	JQ905202	JQ672391	AY563274

Abbreviations of isolates and culture collections: ATCC ATCC ATCC—American Type Culture Collection, Virginia, USA; CBS—Centraalbureau voor Schimmelcultures, Utrecht, Netherlands; MFLUCC—Mae Fah Luang University Culture Collection, Chiang Rai, Thailand; JZB—Culture collection of Institute of Plant and Environment Protection, Beijing Academy of Agriculture and Forestry Sciences (China); ZHKUCC — Culture collection of Zhongkai University of Agriculture and Engineering. Sequences produced in this study are shown in red. T Indicates ex-type/ex-epitype isolates.
